# Supplementary material for: Evaluating modes of influenza transmission (EMIT-2): Insights from lack of transmission in a controlled transmission trial with naturally infected donors
Source: PLoS Pathog. 2026 Jan 7;22(1):e1013153. doi: 10.1371/journal.ppat.1013153 (PMC12799188; doi:10.1371/journal.ppat.1013153)
Supplement: S2 Table — (DOCX) [file ppat.1013153.s006.docx]

### S2 Table. All Donors demographics

|  | **Cohort 24a** | **Cohort 24b** | **Cohort 24c** | **All** |
| --- | --- | --- | --- | --- |
| **Number of participants** | 1 | 1 | 4 | 6 |
| **Female, N (%)** | 1 (100) | 1 (100) | 3 (75) | 5 (83) |
| **Age, mean (SD)** | 25.0 (-) | 23.0 (-) | 20.5 (0.577) | 21.7 (1.97) |
| **Vaccination, N (%)** | 0 (0) | 0 (0) | 2 (50) | 2 (33) |
| **Latino, N (%)** | 0 (0) | 0 (0) | 2 (50) | 2 (33) |
| **Race** |  |  |  |  |
| **Asian, N (%)** | 0 (0) | 1 (100) | 0 (0) | 1 (17) |
| **Black or African American, N (%)** | 1 (100) | 0 (0) | 2 (50) | 3 (50) |
| **White, N (%)** | 0 (0) | 0 (0) | 2 (50) | 2 (33) |
