# Supplementary material for: Evaluating modes of influenza transmission (EMIT-2): Insights from lack of transmission in a controlled transmission trial with naturally infected donors
Source: PLoS Pathog. 2026 Jan 7;22(1):e1013153. doi: 10.1371/journal.ppat.1013153 (PMC12799188; doi:10.1371/journal.ppat.1013153)
Supplement: S4 Table — (DOCX) [file ppat.1013153.s008.docx]

### S4 Table. Summary of Donors cough and sneeze counts during 30-minute breath sample sampling

| Donor | Cough counts,  median(min, max) | Sneeze counts,  median(min, max) |
| --- | --- | --- |
| D24b-1 | 0 (0, 6) | 0 (0, 0) |
| D24c-1 | 0 (0, 0) | 0 (0, 0) |
| D24c-2 | 0 (0, 6) | 0 (0, 0) |
| D24c-3 | 0 (0, 4) | 0 (0, 1) |
| D24c-4 | 5 (0, 16) | 0 (0, 0) |
| All | 0 (0, 16) | 0 (0, 1) |
