# Supplementary material for: Evaluating modes of influenza transmission (EMIT-2): Insights from lack of transmission in a controlled transmission trial with naturally infected donors
Source: PLoS Pathog. 2026 Jan 7;22(1):e1013153. doi: 10.1371/journal.ppat.1013153 (PMC12799188; doi:10.1371/journal.ppat.1013153)
Supplement: S1 Fig — Three Recipients from Cohort 23a re-enrolled in 2024. Two initially joined Cohort 24a, but one was discharged before the study began due to a respiratory infection other than influenza and later re-enrolled in Cohort 24c. Another Recipient from Cohort 23a also joined Cohort 24c. As a result, the total number of distinct individuals who entered quarantine was 27, rather than the summed total (31) from each individual cohort. Note: Influenza Donors were only enrolled in cohorts 24b and 24c; no influenza Donors were enrolled in 23a or 24a. (DOCX) [file ppat.1013153.s009.docx]

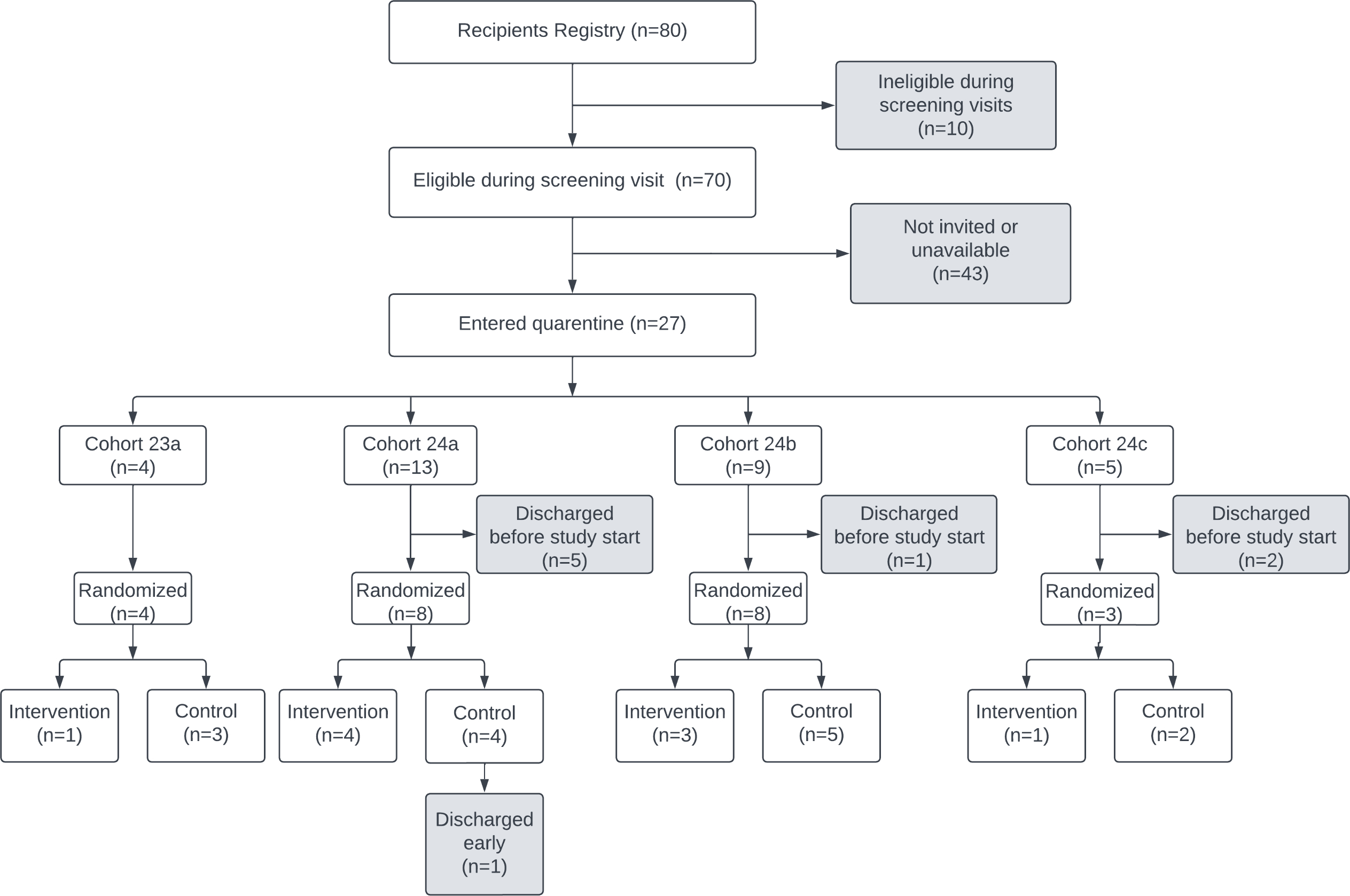


### S1 Fig. Consort diagram for Recipients

Three Recipients from Cohort 23a re-enrolled in 2024. Two initially joined Cohort 24a, but one was discharged before the study began due to a respiratory infection other than influenza and later re-enrolled in Cohort 24c. Another Recipient from Cohort 23a also joined Cohort 24c. As a result, the total number of distinct individuals who entered quarantine was 27, rather than the summed total (31) from each individual cohort.

Note: Influenza Donors were only enrolled in cohorts 24b and 24c; no influenza Donors were enrolled in 23a or 24a.
