# Supplementary material for: Evaluating modes of influenza transmission (EMIT-2): Insights from lack of transmission in a controlled transmission trial with naturally infected donors
Source: PLoS Pathog. 2026 Jan 7;22(1):e1013153. doi: 10.1371/journal.ppat.1013153 (PMC12799188; doi:10.1371/journal.ppat.1013153)
Supplement: S2 Fig — a. “Phone call received” includes inquiries from social media, flyers, television, and other advertisements. b. Many individuals were ineligible due to lack of influenza infection or were unavailable to commit to the quarantine period. c. Ineligibility among screened individuals included symptom onset >48 hours or underlying medical conditions that did not meet eligibility criteria (S3 Text). d. The Donor enrolled in cohort 24a, recruited from among the phone calls received, tested positive for a seasonal coronavirus (229E); no Donors were recruited for cohort 23a. (DOCX) [file ppat.1013153.s010.docx]

**
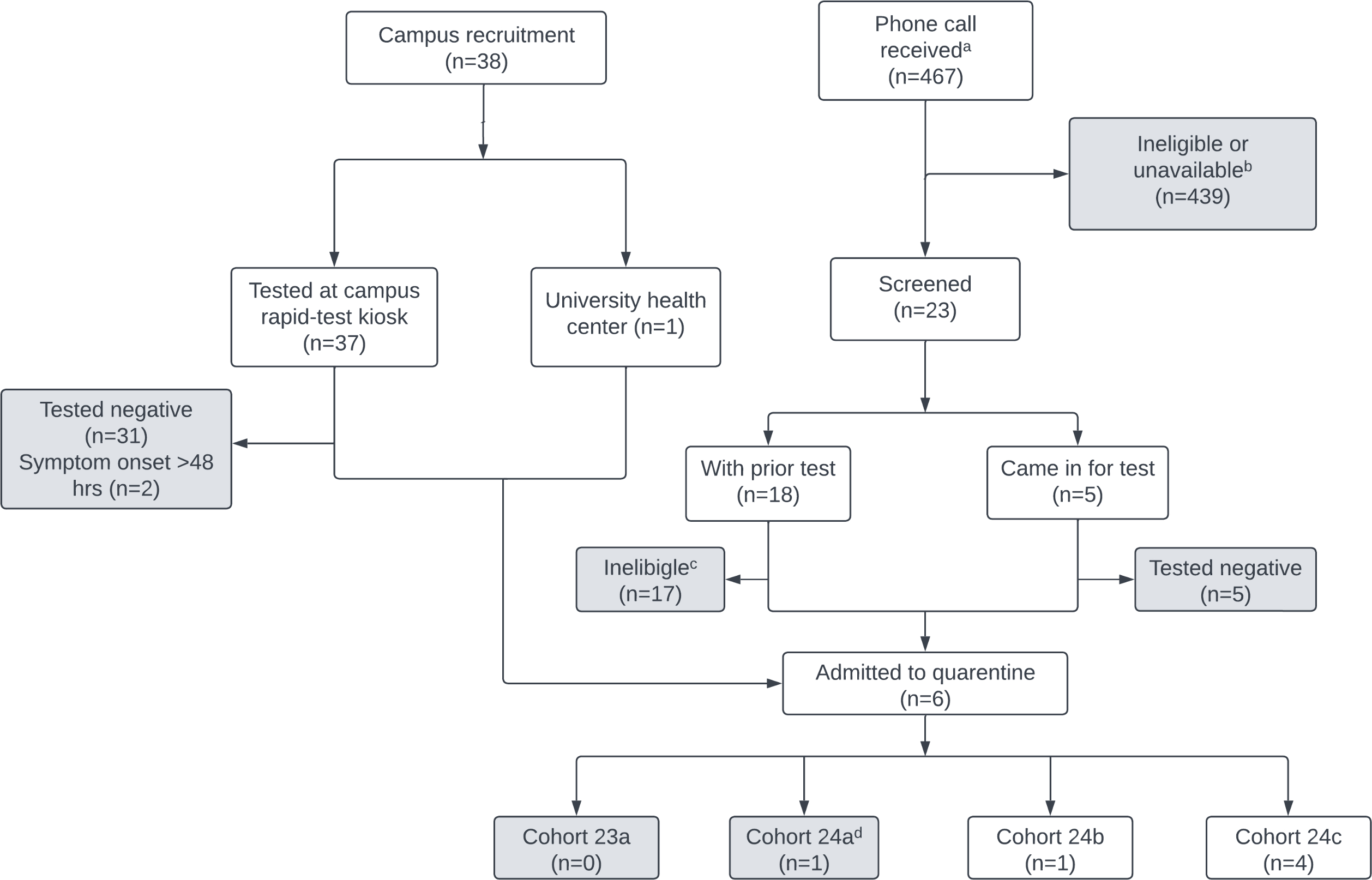
**

### S2 Fig. Consort diagram for Donors

a. “Phone call received” includes inquiries from social media, flyers, television, and other advertisements.

b. Many individuals were ineligible due to lack of influenza infection or were unavailable to commit to the quarantine period.

c. Ineligibility among screened individuals included symptom onset >48 hours or underlying medical conditions that did not meet eligibility criteria (S3 Text).

d. The Donor enrolled in cohort 24a, recruited from among the phone calls received, tested positive for a seasonal coronavirus (229E); no Donors were recruited for cohort 23a.
