# Supplementary material for: Evaluating modes of influenza transmission (EMIT-2): Insights from lack of transmission in a controlled transmission trial with naturally infected donors
Source: PLoS Pathog. 2026 Jan 7;22(1):e1013153. doi: 10.1371/journal.ppat.1013153 (PMC12799188; doi:10.1371/journal.ppat.1013153)
Supplement: S4 Fig — (a) RNA copies from ambient bioaerosol samples collected using NIOSH BC-251 bioaerosol sampling devices, stratified by size fraction (>4 μm, 1–4 μm, and <1 μm). (b) RNA copies (left y-axis) and infectious viral load (FFU, right y-axis) from surface swabs collected from marker pens, microphones, and tablets. Each point represents one sample. Only one surface sample, collected from a marker pen, contained virus detected by dPCR; it was also positive by viral culture. (DOCX) [file ppat.1013153.s012.docx]

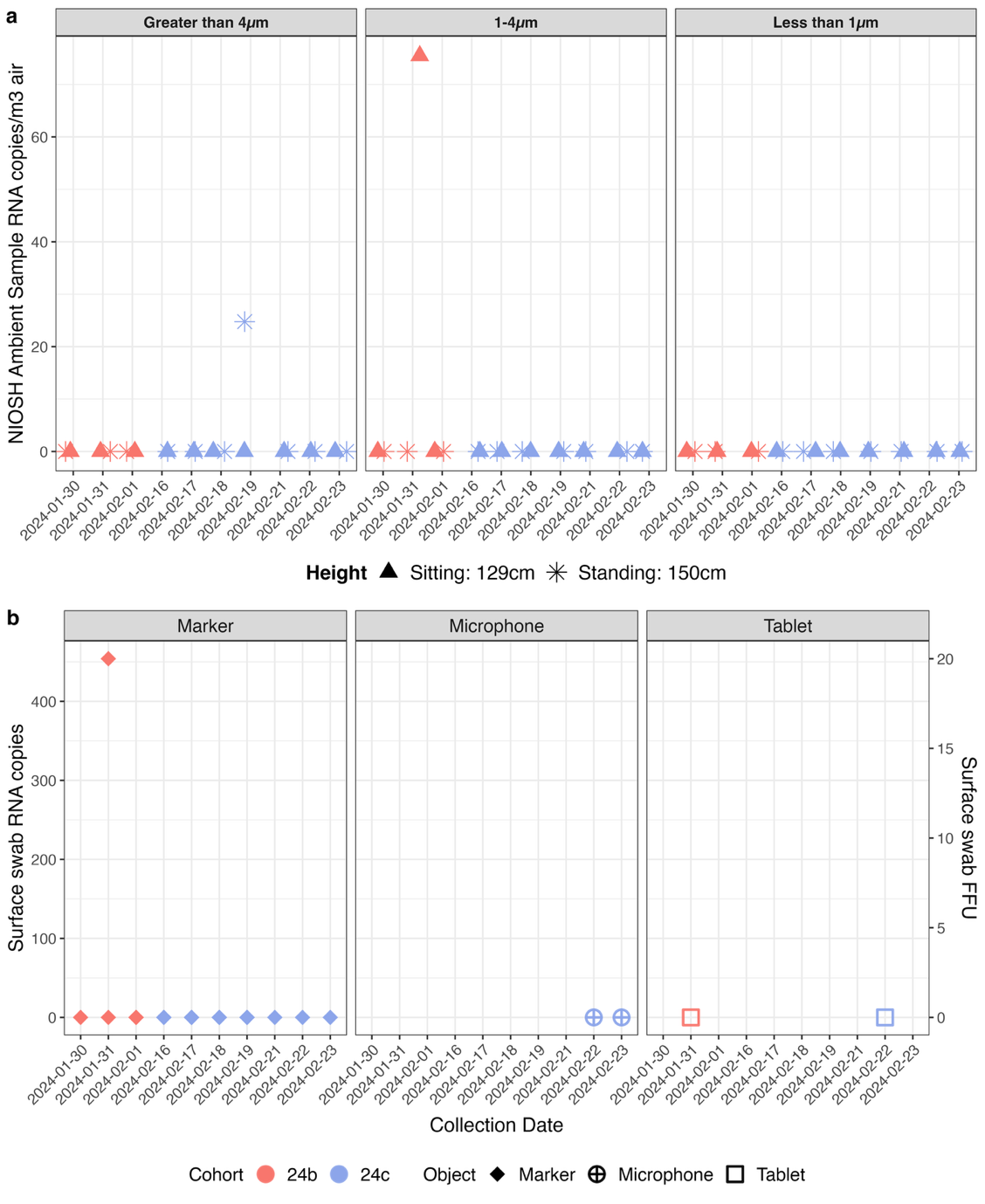


### S4 Fig. Viral load in ambient bioaerosol and surface swab samples

(a) RNA copies from ambient bioaerosol samples collected using NIOSH BC-251 bioaerosol sampling devices, stratified by size fraction (>4 μm, 1–4 μm, and <1 μm).

(b) RNA copies (left y-axis) and infectious viral load (FFU, right y-axis) from surface swabs collected from marker pens, microphones, and tablets. Each point represents one sample. Only one surface sample, collected from a marker pen, contained virus detected by dPCR; it was also positive by viral culture.
