# Supplementary material for: Evaluating modes of influenza transmission (EMIT-2): Insights from lack of transmission in a controlled transmission trial with naturally infected donors
Source: PLoS Pathog. 2026 Jan 7;22(1):e1013153. doi: 10.1371/journal.ppat.1013153 (PMC12799188; doi:10.1371/journal.ppat.1013153)
Supplement: Attachment — Submitted filename: Response to Reviewers-v2.0.1.docx [file ppat.1013153.s016.docx]

Dear Drs. Martínez-Sobrido, Schnell, Bhaduri-McIntosh, and Reviewers,

Thank you for your thorough review and thoughtful comments on our manuscript. We sincerely appreciate your insights and suggestions, which have strengthened the work.

Below we summarize our responses to each comment and indicate the corresponding line numbers in the clean version of the revised manuscript.

Thank you again for the opportunity to revise and improve our manuscript.

Best regards,

Jianyu Lai, Donald Milton, and co-authors

**Reviewers' Comments:**

Reviewer's Responses to Questions

**Part I - Summary**
Please use this section to discuss strengths/weaknesses of study, novelty/significance, general execution and scholarship.

Reviewer #1: In the manuscript by Lai et al ‘Evaluating Modes of Influenza Virus Infection Transmission (EMIT-2): Challenges of a Controlled Human Influenza Virus Infection Transmission Trial (CHIVITT)’, the authors set out to establish a human transmission model using donors with community acquired infections. Prior studies by this team and others using experimentally infected donors have resulted in no transmission, and the authors set out to overcome hurdles with virus strain and ventilation by using a poorly ventilated hotel with naturally infected donors. Unfortunately, of the 4 cohorts the team set out to do, only 2 had both donors and recipients enrolled and even then, no human-to-human transmission of influenza virus was observed. While very disappointing, this observation may reveal something more important about community transmission of influenza viruses. However, the paper, in its current form, fails to analyze the results with this lens and is largely a reporting of negative data. My suggestions to the authors emphasize ways in which the data should be restructured and extended to move the field forward and provide larger insight into influenza virus transmission.

A general comment is that the overall pitch of this manuscript is on the original intent of the project (an intervention study) but this is not what the authors were able to do given the lack of transmission. I would suggest that the authors refrain using language that implies that this study accomplishes this goal (see lines 32-33 abstract, author summary, lines 107-109, and largely in the discussion). This will require a considerable rewrite of the manuscript. The cost of these studies is large and therefore it may be hard to separate out the original intent from the observations that can be most useful for a field when the desired outcome is negative.

Response: We appreciate the Reviewer’s insightful comments. In response we extensively revised the manuscript in an effort to point to lessons learned and future approaches to these studies. This includes a major rewrite to the abstract, summary, parts of the introduction, abbreviation or deletion of much of the material about the interventions, and a nearly compete rewrite of the discussion.

Reviewer #2: In this study, a controlled human influenza virus infection transmission trial was conducted to study its transmission route and transmission dynamics, which contributed to the development and implementation of prevention and control strategies.
However, several areas require attention to ensure the manuscript meets the standards for publication.

Reviewer #3: The study by Lei et al. represents an interesting attempt to experimentally investigate the relative contributions of different influenza transmission modes, airborne, droplet, and contact under controlled conditions. The authors established a quarantine hotel environment to simulate real-world close-contact exposure between donors with community-acquired influenza and healthy recipients.

A key strength of the study lies in its ambitious design, aiming to experimentally replicate influenza transmission in a naturalistic yet controlled environment. The integration of multiple biological sampling methods across personal, ambient, and surface domains enhances the robustness of the environmental virological data. Furthermore, the authors transparently report challenges, including variable donor shedding, pre-existing recipient immunity, and logistical limitations imposed by influenza season timing and recruitment constraints.

A major limitation is the lack of observed transmission to any recipients. This outcome, while informative, limits the ability to draw firm conclusions about the relative importance of different transmission routes. However, this null result is itself valuable, as it highlights key design barriers that must be addressed in future transmission studies. The study’s findings underscore the importance of early-stage donor recruitment, accurate immunity screening, and increasing cohort size to improve transmission observation power.

Overall, the study will be of interest to researchers and readers, and it provides a critical foundation for refining human challenge transmission models to better understand and mitigate influenza spread.

Reviewer #4: This manuscript by Lai and colleagues from the EMIT-2 Study Team analyzes and discusses the challenges of a controlled human Influenza virus infection transmission trial (CHIVITT). The study design involved donors with community-acquired influenza infections and recipients in the quarantine hotel rooms. The comprehensive approach investigated the transmission modes, including analyzing fine and coarse aerosol, mid-turbinate swabs and saliva using RT-PCR and virus culture. There is no seroconversion occurred (recipient got infected) after the controlled exposure during the trial.
The objective of this study has the potential to provide significant insights into viral transmission at community scale and outside lab environment and should attract broader scientific interest. Compared to the previous EMIT-1 study from the study team, the current study includes donors with community-acquired infections rather than challenge infections. The current design offers enhanced comprehensiveness through monitoring and analysis of both environmental samples and biological samples.
While the limitations of this study are acknowledged in the discussion, they significantly impact the interpretation and significance of the results. The authors have conducted power analysis, but the number of the participants are less than the target sample size. The results indicate no recipient infection. It is then unclear to conclude if this outcome is due to the limited number of recipients or reflects other factors on the virus transmission efficiency. Lack of positive recipient may also undermine the anticipated primary outcome to evaluate modes of the transmission.

Reviewer #5: This study presents a controlled human investigation conducted 2023-2024 influenza season to better understand airborne influenza transmission. The authors recruited five influenza-confirmed patients as donors (3 with H3N2 and 2 with H1N1) and exposed them to 11 individuals under monitored conditions. Clinical, virological, and serological data were collected, demonstrating that all five donors had detectable viral RNA, while four of the five had infectious virus in nasal swabs and actively shed viral RNA. However, only one of these donors shed detectable infectious viral particles. Viral RNA was also detected in air samples. The results showed that none of the exposed individuals became infected.

Overall, this is a well-conducted and clearly presented study that illustrates the complexity of influenza transmission in humans and provides valuable data to the field, despite the small sample size and the absence of transmission events. However, several clarifications are needed:

1) The Methods section should include additional detail. For example, the authors need to clarify whether the digital PCR assay targets the M gene, and whether this refers to digital droplet PCR. They also need to provide more explanation on how fluorescent focus units (FFU) were quantified and whether conventional TCID50 or plaque assays (PFU) were also performed. Citation is need to support FFU can be used to detect the infectious influenza virus particles. They need to clarify how the RNA concentration per cubic meter of air was calculated. The ELISA setup needs to be detailed, including which antigens were used.

Response: We revised the supplementary method (S5 Text) to provide more information to address these comments.

- Target gene: We noted that the dPCR assay targeted M gene segment in the Materials and Methods sections of both the main text and supporting information.
- Digital PCR: Qiagen’s QIAcuity One System mentioned in S5 Text was a digital PCR, not droplet digital PCR system. To avoid the confusion, we added this information to the Materials and Methods section.
- FFU quantification: We explained the process of the focus assay in more detail in S5 Text. The use of fluorescent focus assay was previously published and cited, and it was chosen over plaque assay because some seasonal influenza viruses may not produce clearly defined plaques. TCID was not performed.
- ELISA: detailed ELISA methods were provided in the S5 Text. The antigens used were A/Victoria/4897/22 H1 and A/Darwin/6/21 H3.

We also clarified how the RNA concentration per cubic meter was calculated in Lines 497-498 & 501-503: The collected material was extracted into 1 mL of extraction buffer, and viral RNA was quantified from this volume. To calculate RNA concentration per cubic meter of air, we divided the detected the total RNA copy number by the total sampled volume (0.84 m^3^), yielding RNA copies per m^3^ of air.

2) The detection limits for all of these assays need to be presented, and figures showing both viral RNA and infectious particle data should be included to support interpretation.

Response: We expanded the Methods and Supplementary Methods (S5 Text) to include detection limits for assays.

We previously presented viral RNA and infectious viral load as Figures 5 and 6. We updated the figures so that both information is presented in the same figures. Current figure 5 shows the overall viral RNA and infectious viral load in each type of Donor samples, while figure 6 presents their trajectories for each individual Donor, along with positive environmental samples, Donors’ cough counts during 30-min breath sampling and their self-reported total symptom scores.

3) The authors need to clarify whether the two positive samples originated from the same room and the same individual (lines 202–205).

Response: We clarified in the revised Results section (current Lines 221-227) the timing of these ambient sample collections and which Donors were present in the room at the time. We also added a new figure in the SI (S2 Fig) to visualize this information.

4) The color schemes used in the figures could be improved for clarity. The current shades of blue and green, as well as red and purple, are difficult to distinguish—especially in the legends. The authors may consider using different shapes (e.g., circles vs. squares) or line styles to enhance visual separation.

Response: Thank you for the helpful suggestion. We have updated the figures to use the Okabe–Ito color palette, which is designed for accessibility to people with various forms of color vision deficiency. In addition, we incorporated shape and line style differentiation to further improve clarity. Specifically, lines and points now represent RNA (solid) and FFU (dashed) for: breath aerosol <5 µm (solid circle, orange), breath aerosol ≥5 µm (solid triangle, sky blue), mid-turbinate swab (MTS; solid square, green), and saliva (solid diamond, purple).

**Part II – Major Issues: Key Experiments Required for Acceptance**
Please use this section to detail the key new experiments or modifications of existing experiments that should be absolutely required to validate study conclusions.

Generally, there should be no more than 3 such required experiments or major modifications for a "Major Revision" recommendation. If more than 3 experiments are necessary to validate the study conclusions, then you are encouraged to recommend "Reject".

Reviewer #1: Suggestions for improvement:
1. Focus on the last 2 cohorts that had successful enrollment of both donors and recipients. The other cohorts can be acknowledged in the MM and consort diagram but it does not need to be implied that data will be presented from 4 cohorts when only 2 are meaningful.

Response: We have revised Tables 1 and 2 to report demographics only for the two cohorts with successful enrollment of both Donors and Recipients (24b and 24c). The original tables including data from all four cohorts have been moved to the Supporting Information (S1&S2 Tables). Additionally, we now explicitly state early in the Abstract (Lines 35-37) that only cohorts 24b and 24c included Donors with natural influenza virus infections.

We retained the description of Cohorts 23a and 24a to demonstrate the difficulty of performing this kind of study, and to show that despite successful enrollment of Recipients in these two cohorts, we were unable to recruit Donors with natural influenza virus infection.

2. It is meaningful that forward transmission was not observed and some ways to expand on that include providing more information about the exposure events and additional analysis/information as the to the types of activities performed, the behaviors between donors and recipients etc.

Response: We agree and expanded the Results (Lines 159 to 164) and Methods (Lines 457 to 465) to describe activities and Donor–Recipient interactions. We also added S3 Table with event-level activity details and object type. Methods now specify the donor-centered alternating sequence (Donor → Recipient → Donor).

In addition, a few specific comments on the currently presented data are:
a. How many donors and recipients are in each exposure window detailed in Figure 1.

Response: We recreated Figure 1 to indicate the numbers of Recipients and Donors, as well as the introduction timing for each Donor. We also added this information at the event level in our new S3 Table.

b. Lines 152 and 153 indicate two extra exposure events with only recipients, but the timing of these events relative to the transmission events with donors included is not described in the text or indicated on the timeline in Figure 1. If these exposure events are important enough to warrant inclusion, they should be described in more detail.

Response: We agree that these two events without Donors are not important since we did not see primary transmission. We now remove the statement from the revised text.

c. In exposure conditions – include why these parameters are important to measure in the text and provide information about the various exposure window on the graph or activities to put the values into context for the exposure event.

Response: We updated Figure 4, with additional background yellow bands to denote each exposure event windows and vertical ticks to mark exposure start (red) and end (black). We also edited the caption of Figure 4 to explain the importance of these parameters and what they mean in the context of the exposure events.

d. Please note two staff observers when describing the number of occupants in the exposure events in lines 164 and 165. It was not immediately clear why these numbers did not match the number of Donor and Recipient participants.

Response: we edited the sentences (current Lines 179-181) to include this information.

e. Specify the surfaces swabbed and which one was positive (line 210).

Response: we added this information in Lines 232-235. We also added a new S1 Fig to visualize this information.

3. The lack of infectious virus in expelled aerosols is interesting and should be further expanded.
a. Explore the data comparing the H3 infected to the H1 infected?

Response: Thank you for this suggestion. We examined the data and found that all positive exhaled breath aerosol samples, including the single culture-positive sample, were from Donors with H3N2 infections. We briefly noted this finding in the Results section (Line 196 & 201). In a new supplementary figure (S1 Fig), we indicate samples from Donors with H3N2 infections as solid points and those from Donors with H1N1 infections as hollow points so it can be visualized clearly.

b. Did the expelled virus correlate with saliva or nasal swab titers?

Response: We added a supplementary figure (S1 Fig) to depict the correlation of exhaled breath aerosol viral RNA with MTS and saliva samples in our Donors’ samples. We also edited the main text (Lines 212-214) to reflect this finding. Due to a very small sample size, we would not expect to see a significant correlation among these samples. We note that lack of correlation is consistent with our previous paper with a much larger sample size showing that viral load in nasal swabs was not a significant predictor of aerosol shedding (Yan et al., PNAS, 2017).

c. Line 135 states there is evidence indicating that Donor D24c-3 had earlier onset, however this evidence is never provided. Please provide evidence for this statement.

Response: A rapid flu test result submitted subsequently by Donor D24c-3 indicated that they had earlier onset (test date/time greater than 48 hours). We revised the text in Lines 142-144 to indicate this finding.

4. The difficulty with human transmission studies is surprising given the spread in communities. Please expand upon the inclusion and exclusion criteria for both recipients and donors. Address:
a. During donor screening, there was a low probability that a potential subject would be eligible to participate in the study. There is little discussion of the reasons why most subjects were deemed ineligible. These reasons could be useful to the discussion of why carrying out this type of research is difficult.

Response: We have updated Figure 3 and its caption to include additional details regarding ineligibility and screening outcomes. We employed two recruitment strategies for Donors: one through campus-based recruitment, where eligibility was clearly documented, and another via inquiries from social media, flyers, and advertisements. Many individuals who called were ineligible due to the absence of an active influenza infection or were unavailable to participate due to the time commitment required for quarantine. These determinations were made by study staff during phone screening but were not formally recorded. Additional reasons for ineligibility among screened individuals included symptom onset more than 48 hours prior to testing or underlying medical conditions that met our exclusion criteria, as detailed in the Supporting Information (S3 Text).

b. Were antibody levels or recent vaccination used as exclusion criteria for recipients?

Response: Antibody levels and recent vaccination were not used as exclusion criteria for Recipients. We added a sentence to this effect (lines 125-127). This is also reflected in Figures 9 and 10 (antibody levels) and Table 1 (vaccination status). While we had originally planned to include these as exclusion criteria, recruitment challenges necessitated dropping them.

Reviewer #2: The analysis of the results is currently a weak point of the article. The biggest problem with this article is that there is no differential comparison and correlation analysis of the results of several transmission routes. Since it is a study of transmission dynamics and transmission routes, the differences and correlation analyses between samples such as aerosols and surface swabs that highlight different modes of transmission in this study can better reflect which methods of transmission of influenza viruses are predominantly transmitted.

Response: We agree that a clearer comparison across different sample types can enhance the interpretation of potential transmission routes. In response, we have added a new figure (Figure 6) that aligns all collected samples with the cohort timeline, allowing for visual comparison of viral trajectories across exhaled breath aerosols, mid-turbinate swabs, and saliva samples. This provides an integrated view of how these sample types relate to one another over time, particularly around the detection of positive surface swab and ambient aerosols.

To further address the point about correlation analysis, we have added a new S1 Fig that explores the associations between exhaled breath aerosol viral load and viral loads in mid-turbinate and saliva samples.

However, we would like to note that, due to the limited detection in surface swabs (only one positive sample) and the absence of confirmed transmission events during the study period, it would be speculative to draw firm conclusions about the relative importance of different transmission routes. We have therefore taken a cautious approach in interpreting these findings while still presenting the newly included analyses to support transparency and provide a foundation for future research.

As pointed out by Reviewer #1, in the absence of transmission, it makes more sense to focus on what we can learn about influenza dynamics from this study and to reduce emphasis on the intervention. That is now the focus of this paper.

Reviewer #3: 1) The role of pre-existing immunity appears to be important to the lack of transmission, yet the discussion around recipient susceptibility could be expanded. In addition to HAI titres and binding antibody levels, the authors should more thoroughly examine whether mucosal immunity (e.g., sIgA, if available) or T-cell responses might have contributed to protection. I appreciate that new assays can't be added, a deeper discussion of immune correlates and limitations of the current serological data would be valuable.

Response: While our study was limited to serum-based assays due to funding constraints, we agree that other immune mechanisms, such as mucosal immunity, may contribute to Recipient susceptibility. We have revised the Discussion (Lines 399-401) to acknowledge this limitation. Otherwise, we expanded the discussion about susceptibility of middle-aged volunteers to circulating influenza viruses.

2) The limited virus shedding by donors is cited as a key challenge. However, the manuscript would benefit from more detailed shedding profiles, including peak shedding window relative to exposure events and whether donors were within the expected infectious period during interactions. A figure aligning symptoms, viral loads and exposure windows would help contextualise the risk of transmission.

Response: We created a new figure (Figure 6) that presents individual-level trajectories of viral RNA and infectious viral load in Donor samples (mid-turbinate swabs, saliva, and exhaled breath), along with Donors’ cough counts during breath sampling, self-reported symptom scores, and any positive environmental detections, all aligned over the cohort timeline.

Donors were enrolled within 48 hours of symptom onset to capture the early infectious period. As shown in Figure 6, viral loads fluctuated over time, suggesting that several Donors were still near their peak shedding during exposure events. Notably, the only culture-positive breath sample was collected on the Donor’s final day in quarantine, when their breath aerosol viral load was increasing, while nasal and saliva viral loads were declining. This pattern suggests the Donor may have been discharged around or just before their peak aerosol shedding.

3) While viral RNA was detected in aerosols and on surfaces, it would be valuable if the authors could describe whether detected quantities approach infectious doses based on prior literature or other models. Highlighting thresholds of concern would help interpret the biological plausibility of transmission under these conditions, even in the absence of recipient infection.

Response: We revised the discussion (Lines 289-298, and 309-327) to incorporate the estimated RNA copies per infectious quantum from prior literature and compared this threshold to the estimated inhalation exposure based on our whole-room NIOSH aerosol samples. We also added a comparison of our Donors’ exhaled breath viral shedding to that of Donors in the first EMIT study to help understand the low contagiousness of our Donors.

Reviewer #4: (No Response)

Reviewer #5: N/A

**Part III – Minor Issues: Editorial and Data Presentation Modifications**
Please use this section for editorial suggestions as well as relatively minor modifications of existing data that would enhance clarity.

Reviewer #1: 1. There are several potential issues with the data presentation in Figures 5 and 6. A). Some of the y-axis legends could be more informative. It is unclear whether the RNA copies are per dPCR reaction, per sample, per volume unit, or some other unit. Likewise, it is unclear what the denominators for the FFU values are.

Response: Thank you for pointing this out. We have revised the figure caption to clarify the units for both RNA copies and FFU values. Specifically, we now indicate that values are presented per 30-minute sample for breath aerosols, per swab for mid-turbinate swabs (MTS), and per mL for saliva. Because the units differ by sample type, we did not modify the y-axis label directly but instead provided detailed unit information in the caption to avoid confusion.

B). It is unclear why the day values on the x-axis are whole numbers in some panels and non-whole numbers in the Loess plots in panel B. Additionally, it is unclear what the purpose of the Loess smoothed curves is.

Response: The LOESS curves were originally included to illustrate overall trends across Donors; however, given the limited number of samples and the variability in sampling days, we agree that they may be misleading in this context. We have removed the LOESS curves in the updated figures to avoid confusion.

C). Consider making the y-axis scales consistent in panels c, d, e, and f to make it easier to compare the levels in the different sample types.

Response: In the updated version of the figures, we made the y-axis scales consistent for better comparison across sample types.

2. Key details regarding the dPCR assay, including the gene target and sequences, should be included or have a prior publication referenced.

Response: We added more detailed descriptions of the dPCR assay, including its target and citation, in both the Materials and Methods section and S5 Text Supplementary Methods.

We provided more details in the revised supplementary methods (S5 Text) for the dPCR assay.

3. Consort diagrams in Fig 2 and 3 can be combined to demonstrate that cohorts 23a and 24a failed to enroll donors.

Response: While we kept Figures 2 and 3 separate to reflect the distinct recruitment processes for Recipients and Donors, we revised the captions of both figures to clarify that cohorts 23a and 24a did not include any enrolled influenza Donors. In Figure 3, we also shaded the boxes for cohorts 23a and 24a in the Donor Consort diagram to visually highlight the absence of influenza Donors. We hope these additions help clarify the overall study flow.

Reviewer #2: Line 91-93 The author should show the extensive research in introduction.

Response: We revised the sentence (Lines 93-95) to shift the focus from “extensive research” to acknowledging that, despite the recent emphasis on airborne transmission—supported by the WHO’s April 2024 technical report and a recent review article—the relative importance of different transmission modes remains poorly understood. We also cited NIAID’s strategic plan and a recent RFA to further support this point and reflect ongoing efforts to address this research gap.

Result: In line 113, in the time graph of the queue, the author is advised to modify the image to add the corresponding time unit.
This modification will help improve the intuitiveness of the graphical representation。

Response: We have revised Figure 1 to include the time unit on the x-axis. Additionally, we added date annotations to indicate the start date of each cohort and the specific introduction date for each Donor for improved clarity.

Study cohort and study population: In line 125, the concept of IR appears for the first time, and I suggest that the author explain the role of IR when the concept of IR first appears, rather than just in the conclusion part, to increase the comprehensibility and smoothness of the article.

Response: While the roles of IR and CR were originally explained in the Methods section, we have now moved the definition of IR earlier in the Results section (Lines 127–129), where the term first appears, to improve clarity and ensure smoother reading.

Exhaled Breath Aerosol Samples: On line 178, 8.9x103 copies should be changed to 8.9×103 copies.
The symbol "×" is used to denote the multiplication of numbers, which is different from the Roman numeral "x".
There are several places in the article about this symbol, and I recommend that the author review and standardize the unit symbol throughout the manuscript to maintain consistency in the use of units.

Response: Thank you for noticing this. We have updated the symbol throughout to use “×” instead.

Surface Swab Viral Load: In this subsection, it is recommended that the author add a graph on surface swab load. This provides a more visual indication of the propagation strength of the mode of propagation represented by the surface swab.

Response: We added a new S2 Fig for the ambient samples and surface swab samples.

It is suggested that the author add a diagram illustrating the overall experimental results and a model for future epidemic prevention and control.

Response: Thank you for the suggestion. As mentioned earlier, we created a new Figure 6 to align all collected samples with the cohort timeline and provide a visual comparison of viral trajectories across exhaled breath aerosols, mid-turbinate swabs, and saliva samples. The figure also includes positive environmental detections (surface swabs and ambient aerosols), Donors’ cough counts during 30-minute breath sampling, and self-reported total symptom scores. Together, these elements offer an integrated view of the overall experimental results.

Reviewer #3: Line 39: “florescent focus assays”, should be fluorescent focus assay
Line 40: add abbreviation (ELISA)

Response: We updated the typo and added the abbreviation.

Line 44: there is no description what NIOSH is.

Response: Thank you for pointing this out. We clarified in the Methods section that ambient air samples were collected using BC-251 bioaerosol samplers developed by the National Institute for Occupational Safety and Health (NIOSH). To avoid confusion, we have now removed the acronym “NIOSH” from the abstract and refer to the samples simply as “ambient air”, while retaining the full explanation in the Methods section.

Line 351-352: “CRs did not wear face shield or sanitize their hands during the exposure event; they would have been exposed to all modes of transmission”. Was the behaviour of the CRs influenced by their awareness of the infection risk? For example, did they consciously avoid touching their face, compared to typical everyday behaviour in individuals who are not actively considering infection risk? Given the high frequency with which people typically touch their faces per hour, this could be an important factor affecting the likelihood of contact transmission.

Response: We agree that the behavior of CRs may have been influenced by their awareness of potential infection risk, which could have affected the likelihood of contact transmission. We added this as a limitation in our revised manuscript (Lines 394-399) and suggested that future CHIVITT studies consider incorporating behavioral requirements for CRs, such as touching their face at regular intervals, to ensure consistent exposure to all transmission modes.

Additionally, during the EMIT-2 study, we collaborated with engineering colleagues to develop a computer vision system that analyzes participant behavior using video recordings from the exposure events. These data are currently under analysis and are being incorporated into a separate manuscript that is under review.

Line 394: “score throat” should be sore throat

Response: We corrected the typo.

Figure 5 caption: a) put abbreviation for mid-turbinate swabs (MTS), “aaliva” should be saliva

Response: We added the abbreviation and corrected the typo in the caption.

Reviewer #4: 1. The authors have mentioned that compared to EMIT-1, EMIT-2 recruited symptomatic community-acquired donor to achieve expected SAR. However, the current study lacking statistically significant data, could not provide a direct comparison or evaluation of this under this experimental setup. In the future study, increasing the number of donors to reach the expected power is necessary. Given the inherent limitations in recruiting sufficient community-acquired donors within specific timeframes and given periods that possibly missing the peak viral loads, will the authors consider alternative ways like those in EMIT-1 to increase the number?

Response: EMIT-1 used Donors infected via intranasal inoculation, several studies, including our prior work, have shown that this method leads to milder illness and substantially less exhaled breath aerosol viral shedding compared to community-acquired infections. Therefore, we do not plan to reintroduce intranasal inoculation in future studies. However, aerosol inhalation challenge models may offer a promising alternative that better mimics natural infection while enabling more controlled study design. Other groups are pursuing this approach. However, it still entails using a lab-adapted (often egg-adapted) virus. Therefore, we suggest alternatives to enhance the likelihood of observing transmission as described in the extensively revised discussion.

2. The authors mentioned that lower overall contagiousness such as less cough limited the current results. However, again due to the insufficient statistical power, this could not be statistically tested from the current study. Is it feasible or ethically acceptable to establish and request a minimal number of coughs for certain donors during the exposure in comparison to the exposure where cough barely occurred.

Response: We agree with the concern regarding statistical power within the current study. However, over the course of testing over 200 influenza cases, we do have statistical power and point to those other studies in the revised Discussion.

Regarding the possibility of requiring a minimum number of coughs, this is indeed an interesting consideration. While implementing such a requirement may raise feasibility and ethical questions, we will explore this further during the development of future CHIVITT protocols to improve consistency in Donor contagiousness and exposure conditions. We think that focusing on cough as a criterion for Donor recruiting may be a promising approach.

3. Figure 1 and 4 require detailed captions. It would be helpful and clear to indicate the exposure event periods in Figure 4 and label the recipients who have received the vaccinations in Figure 9 and 10. Typo in Figure 5: saliva

Response: We recreated Figure 1 and 4 to indicate the exact timing of exposure event and Donor introduction. We also edited the caption to provide more detailed information.

Figure 9&10: We revised Figures 9&10 to add Recipient vaccination information.

We corrected the typo in our updated Figure 5 and caption.

Reviewer #5:  See summary section.
