# Supplementary material for: Evaluating modes of influenza transmission (EMIT-2): Insights from lack of transmission in a controlled transmission trial with naturally infected donors
Source: PLoS Pathog. 2026 Jan 7;22(1):e1013153. doi: 10.1371/journal.ppat.1013153 (PMC12799188; doi:10.1371/journal.ppat.1013153)
Supplement: Attachment — Submitted filename: 2nd_Response to Reviewers-v0.0.7.docx [file ppat.1013153.s017.docx]

Dear Drs. Martínez-Sobrido, Schnell, Bhaduri-McIntosh, Malim, and Reviewers,

We thank you again for the opportunity to revise our manuscript. We are grateful to the reviewers for their continued thoughtful feedback and appreciate the constructive comments provided during this second round of review.

Below, we detail our responses to the remaining comments (Part III Minor Issues) and indicate the corresponding changes made in the revised manuscript (clean version line numbers provided).

Thank you again for your time and consideration.

Best regards,

Jianyu Lai, Donald Milton, and co-authors

**Part III – Minor Issues: Editorial and Data Presentation Modifications**

Reviewer #1: A few suggestions remain, mostly for increased readability and topics to cover in the discussion.
1. The title does not accurately describe the results, consider revising.

Response: We agree that the original title did not fully capture the emphasis of our current manuscript. In our last revised version, we have updated the title from “Challenges of a Controlled Human Influenza Virus Infection Transmission Trial” to “Insights from a Controlled Human Influenza Virus Infection Transmission Trial” to more accurately reflect the discussion and findings presented in the paper. Reviewer #1 mentioned the original title in comments in Part I. Therefore, we suspect that they overlooked the change already made and hope that the “Insights” title addressed their concern.

2. Figure inclusion and structure:
a. The consort diagrams (Fig 2 and 3) are not very meaningful (expect that recruitment from campus testing kiosk was imperative for identifying eligible donors). Consider moving to the supplement.

Response: We moved both CONSORT diagrams to the supplementary as current S1 & S2 Figs.

b. The S1, S2, S3, and S4 text are confusing. S1 text includes many of the authors – why is this a separate list? Also it is not referenced anywhere that I could tell. S2 text should be in the acknowledgments and S3 and S4 text should be combined into one document.

Response: We appreciate the reviewer’s comments on the supplemental texts.

S1 Text contains the full membership of the EMIT-2 Study Team, which is referenced in the author byline as “for the EMIT-2 Study Team”. This study involved a broader group of collaborators, some of whom contributed to the original proposal and study design but were not directly involved in the reported experiments or producing this particular manuscript. Conversely, some authors contributed significantly to the current analysis and writing but were not part of the broader Study Team. To improve clarity and ensure proper attribution, we added a footnote to the author line stating: “^†^A complete list of EMIT-2 Study Team members is provided in S1 Text”. We are happy to revise this further at the editor’s discretion.

S2 Text contains extended acknowledgments and is explicitly referenced in the Acknowledgments section of the manuscript: “A complete list of contributors can be found in S2 Text.” Given the length of this list, we felt it was appropriate to include in the supplement, but we defer to the editor if it should instead be incorporated into the main text.

S3 and S4 Texts have now been combined into a single supplemental file as suggested (S3 Text. Eligibility Criteria).

3. Correlation graphs in figure S1:
a. The regressions with the aerosol >5 micron are driven by a single positive point and thus not accurate.

Response: We agree that these comparisons do not show meaningful correlation, and we now emphasize this more clearly by updating the figure title to “Lack of correlation between Donor exhaled breath aerosol viral RNA and matched MTS or saliva samples”. We retained the regression lines for visual consistency across panels, but interpretation in the text reflects that no significant correlation was found.

b. Please add more descriptive labels to the axis.

Response: We revised the axis labels to clearly indicate both the sample types and units (e.g., “Breath aerosol ≥5 µm (log10 RNA copies)” and “MTS (log10 RNA copies)”), so that the meaning of each panel is apparent without needing to refer to the figure caption.

4. The three reasons given for the lack of transmission stimulate the following topics that could be expanded in the discussion:
a. Typical indoor environmental conditions (temp, humidity, and ACH) where transmission is known to occur.

Response: We extended the discussion (Lines 361–380) to include literature on the impact of indoor environmental conditions on viral survival and transmission.

b. The heterogeneity in expulsion of viruses into the air. This is mentioned a bit, but beyond the work done by this team, SARS-CoV-2 controlled infections have revealed a heterogeneity in expulsion of virus-containing aerosols.

Response: We added additional discussion (Lines 333–341) on the heterogeneity of viral aerosol emissions. Since our study used community-acquired cases, we cite prior findings from naturally infected individuals, as well as recent human challenge studies that support the presence of substantial inter-individual variability in aerosol shedding.
